# The clinical Spectrum of Viridans Group Streptococci infections in paediatric patients at a tertiary hospital

**DOI:** 10.4102/sajid.v39i1.563

**Published:** 2024-04-29

**Authors:** Nkosinathi S. Shongwe, Fikile C. Mabena, Jeannette Wadula, Karen Petersen

**Affiliations:** 1 Department of Paediatrics and Child Health, Faculty of Health Sciences, University of the Witwatersrand, Johannesburg, South Africa; 2 Department of Paediatrics and Child Health, Chris Hani Baragwanath Academic Hospital, Johannesburg, South Africa; 3 Department of Clinical Microbiology and Infectious Diseases, Faculty of Health Sciences, University of the Witwatersrand, Johannesburg, South Africa; 4 Department of Clinical Microbiology and Infectious Diseases, Chris Hani Baragwanath Academic Hospital, Johannesburg, South Africa

**Keywords:** Viridans Group Streptococcus, antibiotics, susceptibility, clinical presentation, organism

## Abstract

**Background:**

Viridans Group Streptococci (VGS) are often considered organisms of low virulence; however, infection can result in clinically significant sepsis and life-threatening complications in paediatric patients.

**Objectives:**

This study aimed to describe the spectrum of clinical presentation of VGS bacteraemia in paediatric patients, to analyse risk factors, and to describe the antibiotics resistance patterns of VGS.

**Method:**

Cultures of VGS in paediatric patients admitted to Chris Hani Baragwanath Academic Hospital in 2019 were identified through National Health Laboratory Service. Data were extracted from archived clinical records and analysed. Sepsis scores were calculated at the time of bacteraemia.

**Results:**

A total of 133 cultures were identified; 64 (48.1%) polymicrobial cultures and no records 4 (0.03%) were excluded; 65 (48.9%) were analysed. The median age was 1.5 months (range 0.03 to 168, interquartile range [IQR]: 0.3–13.25), 27/65 (42%) were neonates. The median duration of hospitalisation was 7 days (IQR: 3–21). The commonest diagnoses were neonatal sepsis 30.8% (*n* = 20) and pneumonia 28% (*n* = 18). The systemic inflammatory response syndrome (SIRS) score was ≥ 2 in 57% (16/28) patients; paediatric sequential organ failure assessment (pSOFA) score was > 2 in 10/24 (42%). Fifty-seven (88%) patients were discharged; three (5%) required ICU admission and 8/65 (12.3%) died. Malnutrition was present in 50% of patients who died. Cephalosporins and penicillin had sensitivity of 89% and 55%, respectively.

**Conclusion:**

Viridans Group Streptococci bacteraemia was common in neonates, and pneumonia was a common presentation in this cohort. The VGS bacteraemia was associated with morbidity and deaths in this cohort.

**Contribution:**

The VGS should be considered a significant organism when cultured from sterile sites and routine antibiotic susceptibility testing should be performed. Prospective studies are recommended.

## Introduction

Viridans Group Streptococci (VGS) are Gram-positive cocci in chains^1,2^ with at least 30 recognised species.^2^ They are a heterogenous group consisting of five major groups, with multiple subspecies: *Streptococcus mitis, Streptococcus salivaris, Streptococcus anginosus* (*S. milleri*), *Streptococcus mutans* and *Streptococcus bovis* (group D Streptococcus) group.^3^ They are part of the normal flora of the oropharyngeal, urogenital, gastrointestinal tracts^2^ and skin.^4^ When cultured from sterile sites, the question of their pathogenicity often occurs.^4^ Still largely considered to be a contaminant, it can be life-threatening in all patients^1,5^; as many as 32% of isolates have been shown to be of clinical significance.^6^ It can cause invasive disease in immunocompromised paediatric patients,^5^ such as HIV-infected patients, neonates and cancer patients undergoing chemotherapy or stem cell transplant.^2,7,8^ There is very limited literature on this organism other than in patients with malignancies undergoing chemotherapy or stem cell transplant. It was the third most common organism cultured in neonates in a study in Norway.^9^ Of note, clinical associations of VGS bacteraemia include infective endocarditis, central venous catheter infections, aspiration pneumonia, spontaneous bacterial peritonitis and rarely other infections such as meningitis, otitis media, sinusitis and dental infections.^10^

Methods to determine if VGS cultures are true pathogens or contaminants include the following: (1) the number of positive blood cultures in a set, (in true bacteraemia there are often multiple positive cultures of the same organism),^11^ (2) the time to positivity of cultures (cultures which become positive after 3–5 days are more likely to be contaminants),^12^ and (3) clinical features such as fever, hypothermia, low or high white cell count and hypotension predict true infection as opposed to contaminant.^13^

There is ongoing debate on the definition of sepsis. The third consensus for sepsis that was published in 2016 defines sepsis as life-threatening organ dysfunction caused by a dysregulated host response to infection.^14^ Clinical sepsis scores may be used as indicators of sepsis in patients. The systemic inflammatory response syndrome (SIRS) score requires two of four criteria and proven infection, the criteria being: (1) fever or hypothermia, (2) tachycardia, (3) tachypnoea and (4) leucocytosis or leukopenia.^14^ Infection is defined by laboratory documentation of a pathogen by positive culture, tissue stain or polymerase chain reaction (PCR) test, and clinical syndrome associated with a high probability of infection.^15^ The paediatric sequential organ failure assessment (pSOFA) is a better predictor for mortality and has a higher sensitivity for severe infection compared to SIRS.^16^ The pSOFA score has six organ-specific criteria, with each criterion scored from 0 to 4 according to severity with a maximum score of 24. The scoring criteria are (1) renal function (creatinine), (2) cardiovascular (mean arterial pressure), (3) respiratory (PaO2:FiO2 or SpO2:FiO2), (4) haematological (platelets), (5) hepatic (bilirubin), and (6) neurological (Glasgow Coma Scale). The pSOFA score showed excellent discrimination for in-hospital mortality in a general PICU (paediatric intensive care unit) population and in the subgroup of patients with suspected or confirmed infection.^17^ However, pSOFA is not well validated especially for patients outside the PICU setting.^12^

Biomarkers are often used in the diagnosis of sepsis, but there are limitations. C-reactive protein (CRP) as a sepsis biomarker has poor specificity to differentiate between bacterial, viral, and non-infectious inflammatory conditions.^15^ It is useful when used in combination with other biomarkers and to assess response to therapy. White blood cell (WBC) count increase is indicative of inflammation and infection.^18^ However, both WBC count and CRP can be altered in several clinical conditions, such as any blood disorders and in inflammatory non-infective disorders. In addition, the WBC count could be normal or even decreased in some cases of sepsis. Thus, total WBC has a poor specificity, which limits its usefulness as a biomarker of sepsis.^18,19^ The test performance of total WBC count has been variable, with the reported sensitivities and specificities for the commonly used total WBC count threshold in paediatric patients of greater than 15 × 10^9^ WBC/L ranging from 38% to 86% and 53% to 85%, respectively.^20^ The normal range of WBC count in neonates is often higher at 9 to 30 × 10^9^ WBC/L.^21^

Viridans Group Streptococci were previously considered to be susceptible to penicillin, but recent studies have shown an increase in resistance globally. Some studies have found 20% resistance to penicillin.^22^ In a South African study, they found that there was up to 38% resistance of VGS to penicillin.^23^ Tetracycline resistance has been reported as 41% in South Africa^24^ and as high as 70% in Taiwan.^22^ Resistance to fourth-generation cephalosporins ranged from 14% to 34%.^17^ Prior use of beta lactam antibiotics was the only factor significantly associated with cephalosporins resistance. Vancomycin, imipenem, ceftriaxone and cefotaxime had the best susceptibility in a South African study of 211 VGS isolates from blood cultures collected from three Johannesburg hospital laboratories.^23^ Vancomycin had 97% susceptibility in a study in the United States.^24^ Cancer patients were also found to be more likely to culture resistant VGS species, with trimethoprim-sulphamethaxazole resistance at 36% in this group of patients compared to 17% in the rest of the population.^24^

The aim of this study was to determine the clinical spectrum of presentation and clinical significance of VGS infections in paediatric patients using sepsis scores and biomarkers and to determine the resistance patterns of VGS in these patients.

## Methods

This was a retrospective descriptive study for the period 01 January 2019 to 31 December 2019. All paediatric patients from birth to 14 years of age admitted to the Chris Hani Baragwanath Academic Hospital (CHBAH) during this period and who cultured VGS from sterile bodily fluids were considered eligible. Children with polymicrobial cultures on the same sample and those whose clinical records could not be traced were excluded from the study. Lists of positive cultures were obtained with permission from the National Health Laboratory Service (NHLS) microbiology department. Archived inpatient files and electronic discharge summaries were retrieved with permission to obtain clinical data. Information on susceptibility of the organisms was obtained from NHLS. Systemic inflammatory response syndrome and pSOFA scores were calculated for each patient at the time of positive culture. A CRP value of 10 mg/L or more was suggestive of infection. Data were analysed using Statistica.

Testing and speciating of VGS at NHLS was performed using the Clinical and Laboratory Standards Institute (CLSI) guidelines.^25^ When Gram-positive cocci in chains and/or pairs on gram stain was identified, it was plated out onto 5% blood agar and incubated for 18 h – 24 h. The plate was observed for the type of haemolysis; VGS are generally alpha-haemolytic. The isolate was tested for susceptibility to optochin and the routine Gram-positive antibiotic discs using the Kirby-Bauer disk diffusion method (*S. pneumoniae* is susceptible where VGS are resistant). Speciation was performed using the MicroScan Gram-positive panel if it was requested by the treating clinician.

## Definitions

- Underweight: classified according to World Health Organization (WHO) growth chart as weight for age less than –2 standard deviation (s.d.).
- Stunted: classified according to WHO growth charts as height for age Z score less than –2 s.d.
- Wasted: classified according to WHO growth charts, weight for height Z score less than –2 s.d.^26^
- Nosocomial sepsis: VGS isolated in patients with sepsis more than 48 h post admission.

## Results

**FIGURE 1:**
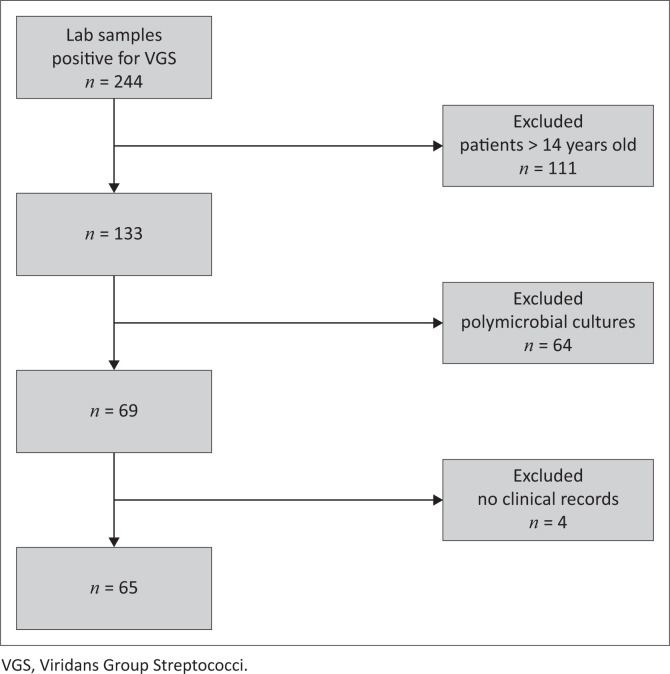
Flow diagram of cases.

The VGS was cultured in 133 specimens from paediatric patients during the study period. Sixty five (65/133, 48.9%) patients were included in the study after excluding polymicrobial cultures 64/133 (48.1%) and those with no clinical records 4/133 (3%), Figure 1.

There were 35 (53.8%) female patients. The median age of the patients was 1.5 months (interquartile range [IQR]: 0.2 to 13.3), and 27 (41.5%) patients were neonates. Ten (15%) patients were HIV exposed with one HIV infected. The HIV-infected patient was 6 weeks old, newly diagnosed and antiretroviral treatment naive. Sixty-two (95.4%) patients were HIV uninfected. Four patients (6.2%) were oncology patients receiving chemotherapy. The median weight for age (WFA) Z score in 61/65 was –1.0 (IQR: –2.0 to 0.0); 19 (29.2%) patients were stunted, and median weight for height (WFH) in 51/65 was 0.0 Z score (IQR: –1.5 to 0.0) with 12 (18.5%) patients were wasted (Table 1).

**TABLE 1:** Patients’ clinical demographics.

Variable	*n*	%	Median	IQR
**Gender**				
Male	29	44.6	-	-
Female	35	53.8	-	-
Unknown	1	1.5	-	-
**Age (months)**	-	-	1.5	0.2–13.25
Neonates (≤ 28 days)	27	41.5	-	-
Infants (> 28 days to ≤ 1 year)	19	29.2	-	-
Children (> 1 to ≤ 14 years)	19	29.2	-	-
**HIV status**				
Uninfected	62	95.4	-	-
Unknown	2	3.1	-	-
Infected	1	1.53	-	-
**Neonatal weight classification**				
Extremely low birth weight	3	11.1	-	-
Very low birth weight	3	11.1	-	-
Low birth weight	3	11.1	-	-
Normal birth weight	18	66.7	-	-
**WFA z-score**				
Normal	24	63.2	-	-
Underweight	11	28.9	-	-
Unknown	3	7.9	-	-
**HFA z-score**				
Normal	16	42.1	-	-
Stunted	14	36.8	-	-
Unknown	8	21.1	-	-
**WFH z-score**				
Normal	23	60.5	-	-
Unknown	8	21.1	-	-
Wasted	7	18.4	-	-
**Clinical diagnosis**				
Neonatal sepsis	20	30.8	-	-
Pneumonia	18	27.7	-	-
Nosocomial sepsis	10	15.4	-	-
Acute gastroenteritis	8	12.3	-	-
Malignancy	4	6.2	-	-
Meningitis	2	3.1	-	-
Submandibular abscess	1	1.5	-	-
Tonsillitis	1	1.5	-	-
Seizures	1	1.5	-	-
**Outcomes**				
Discharged	57	87.7	-	-
Deaths	8	12.3	-	-
Admitted ICU	3	4.6	-	-

WFA, weight for age; HFA, height for age; WFH, weight for height; ICU, intensive care unit; IQR, interquartile range.

The most common clinical diagnoses were neonatal sepsis in 20 (30.8%) and pneumonia in 18 (27.7%) patients. Nosocomial sepsis and acute gastroenteritis (AGE) occurred in 10 (15.4%) and 8 (12.3%) patients, respectively (see Table 1). The median duration of hospital stay was 7 days (IQR: 3 to 21). Viridans Group Streptococci were isolated from blood cultures in 62 (95.3%), 2 (3.1%) from cerebrospinal fluid (CSF) and 1 (1.5%) from abscess aspirate. The median time to positivity of the blood cultures was 12.5 h (IQR 8 to 20). The VGS was cultured once only for all patients. Fifteen (55.6%) of the neonatal cultures were performed in the first 72 h of life. CRP was elevated within 48 h of culture in 53.7% (22/41) with a median value of 10 (IQR: 1 to 27; range 0 to 304). In neonates, the median white cell count was 12.08 (IQR: 8.9 to 14.9) and for children post-neonatal period 10.2 (IQR: 7.58 to 15.4) (see Table 2). Five (7.7%) of the patients in this study were neutropenic (neutrophil count < 1.5 × 10^9^) of which 2/5 (40%) were oncology patients receiving chemotherapy with nosocomial neutropenic sepsis and one was a neonate with neonatal sepsis. All the neutropenic patients survived the sepsis episode. Viridans Group Streptococci meningitis occurred in two infants, both were hospital acquired, one died in ICU and the other survived to discharge. Three patients had presumed meningitis with VGS bacteraemia and CSF chemistry suggestive of meningitis, but the organism was not cultured in the CSF. Antibiotic susceptibility was performed in 57/65 (87.79%) patients. Most of the results did not have minimal inhibitory concentration (MIC) susceptibility testing, which may result in inaccuracies on the susceptibility results. Antibiotic susceptibility for third generation cephalosporins was performed by disk diffusion testing, 50/56 (89%) were susceptible; five (8.9%) resistant and one displayed intermediate susceptibility. Penicillin resistance was 45.8% (5/11) in isolates, which had MIC testing. A total of 10/65 (15.4%) of the cultures were tested for vancomycin and were all susceptible.

**TABLE 2:** Laboratory investigations.

Variable	Neonates					Others > 28 days				
	*n*	%	Median	IQR	Range	*n*	%	Median	IQR	Range
**Specimen type**										
Blood culture	27	100	-	-	-	35	92.1	-	-	-
CSF culture	0	0	-	-	-	2	5.3	-	-	-
Abscess aspirate	0	0	-	-	-	1	2.6	-	-	-
Time to positivity of cultures (hours)	-	-	10	7–17	3–82	-	-	15	10–20	3–42
Leucocytes (×10^9^/L)	-	-	12.1	8.9–14.9	-	-	-	10.2	7.58–15.4	-
Neutrophils (×10^9^/L)	-	-	5.0	2.45–7.96	-	-	-	4.39	2.77–7.28	-
Lymphocytes (×10^9^/L)	-	-	4.28	3.15–5.78	-	-	-	3.88	2.67–5.17	-
Platelets (×10^9^/L)	-	-	260	192–341	-	-	-	312	201–462	-
CRP (mg/L)	-	-	10	1–27	0–42	-	-	12	1–51	0–304
**CSF (*n* = 26)**										
Normal	10	-	-	-	-	11	-	-	-	-
Probable meningitis	1	-	-	-	-	2	-	-	-	-
Confirmed meningitis	0	-	-	-	-	2	-	-	-	-

CSF, cerebrospinal fluid; CRP, C-reactive protein; IQR, Interquartile range.

The SIRS score could be assessed for 28 (43%) patients; mean SIRS score was 1.7, range 0 to 4. Sixteen (57.1%) had a significant SIRS score of two or more. The pSOFA could be calculated in 28 (43%) patients; median score was 1 (IQR: 0 to 4). Severe complications occurred in 10 (15.4%) of the patients; three required intensive care unit (ICU) admission and 8/65 (12.3%) patients died. Of the patients who died, 4/8 (50%) had pneumonia and 4/8 (50%) had nosocomial sepsis (Table 3). The cultures of the patients admitted to the ICU were all performed at the time of the episode of sepsis requiring the ICU. Antibiotic use at the time of the VGS bacteraemia was documented in 32 (49.2%) patients. The median duration of antibiotics use was 5 days (IQR: 5 to 7). Table 3 shows the characteristics of the patients who died.

**TABLE 3:** Clinical and laboratory characteristics of deaths associated with Viridans Group Streptococci bacteraemia.

Patient number	Age category†	Gender	Nutrition‡	HIV status	Diagnosis	Underlying condition	CRP	WBC	SIRS score	pSOFA	Antibiotic susceptibility	Empiric antibiotic used	Time interval in days (culture to outcome)	Time to positivity of culture (hours)
1	Neonate	F	Underweight	Neg	Pneumonia	Premature	1	8.73	2	8	Sensitive	Ampicillin/gentamycin	7	10
2	Neonate	M	Underweight	Neg	NOS	Premature	19	3.13	NC	NC	Resistant ampicillin	Unknown	16	12
3	Neonate	F	Normal	Neg	NOS	DiGeorge/CHD	23	33.8	NC	NC	Not done	Unknown	19	10
4	Neonate	F	Normal	Neg	NOS	Spina bifida	8	11.8	NC	NC	Not done	Unknown	10	6
5	Infant	M	Underweight	Neg	Pneumonia	Trisomy21/CHD	1	9.95	2	5	Resistant cefotaxime	Meropenem	1	8
6	Infant	F	Underweight	Pos	NOS	BPD	102	8.96	0	5	Sensitive	Meropenem/vancomycin	44	CSF (no time stated)
7	Infant	F	Underweight	Neg	Pneumonia	Premature	18	7.32	NC	NC	Sensitive	Unknown	1	12
8	Child	F	Normal	Neg	Pneumonia	Cerebral palsy	300	15.4	NC	NC	Sensitive	Unknown	1	19

CRP, C-reactive protein; WBC, white blood cell; SIRS, systemic inflammatory response syndrome; pSOFA, paediatric sequential organ failure assessment; Neg, negative; Pos, positive; NOS, nosocomial sepsis; CHD, congenital heart disease; BPD, bronchopulmonary dysplasia; NC, not calculable (missing data); F, female; M, male.

† , Neonate, infant child;

‡ , Normal underweight stunted wasted.

## Discussion

The clinical significance of VGS bacteraemia in immunocompetent paediatric patients has not been documented before at this site. This study describes the clinical spectrum and outcomes in paediatric patients with VGS bacteraemia and the antibiotics resistance patterns of the organism over a 1-year period. Except for the neonates and oncology patients on chemotherapy, this study included mostly immunocompetent children; HIV infection and malnutrition were not the predominant patient profile.

In this cohort, neonatal sepsis was the most common clinical presentation; this is similar to other studies,^8^ and two from the same site.^27,28^ Mangeni et al. reported VGS was the most common isolated bacteria in neonates presenting from home with neonatal sepsis.^28^ In a study by Velaphi et al., the VGS was reported to be the most common bacterial cause of early onset neonatal sepsis after Group B streptococcus.^27^

Viridans Group Streptococci sepsis in neonates is most likely because of their immunocompromised state.^29^ The VGS was previously thought to not be part of the normal flora of the newborn’s skin;^7^ however, recent studies show term and preterm infants can be colonised at birth.^30^ In our cohort, pneumonia was the second most frequent clinical manifestation; this clinical manifestation has been documented in other studies.^8,31^ Few cases of viral AGE resulting in secondary VGS bacteraemia have been reported.^32,33^ We could not find any literature reports of VGS as primary cause of AGE. Also, we could not determine in our study whether the AGE was primary or secondary. In a large multicentre study in America, *Streptococcus* species accounted for 5.9% of nosocomial infections with the most common being VGS. Contrary to other reports,^7,8^ the patients with oncological conditions recovered from VGS bacteraemia. However, this should be interpreted with caution because of the small sample size and the exclusion of polymicrobial cultures from this study. Similarly, the absence of cases with infective endocarditis may be explained by multiple possibilities, including missed diagnosis, small sample size, decreased prevalence rates of the disease, and the use of prophylactic antibiotics for dental procedures.^34^ Risk factors for VGS bacteraemia have been described in neutropenic patients including mucositis, use of proton pump inhibitors and previous use of cotrimoxazole^2^; these data were not collected in this study.

The presence of the organism in pure culture, the clinical findings, and time to positivity of less than 24 h support true infection. However, the CRP and WBC as biomarkers of infection were not routinely elevated in this cohort. The limitations of these tests have been discussed before.^16,18^ However, it is possible that a proportion of the cultures may represent contamination. The sepsis scores could not be calculated for some patients because it requires tests which were not performed or documented; this is a limitation of its use in clinical practice especially in developing countries.^35^ The same study found pSOFA to be a better predictor of death than SIRS score.^35^ In our cohort, the pSOFA and SIRS score yielded different results in patients in which it could be calculated; the SIRS score suggested sepsis in most of the patients, but the pSOFA score was not suggestive of sepsis. The invasiveness of the organism as shown by the CSF results also supports true infection.

Some studies have reported a median case fatality rate of 10% (range 0% to 50%).^8^ In this study, 12% of the patients died. As some of the deaths recorded in our study occurred a considerable time after the organism was cultured, not all of them could be definitively attributed to VGS bacteraemia. Prematurity, malnutrition and underlying chronic illnesses could be contributary factors in the causation of death.

Although there were few patients on whom penicillin MIC testing was performed using the Etest method, the high rate of penicillin resistance compared to previous reports from South Africa^23^ is of concern, particularly since this is often used as the first line antibiotic. We recommend routine antibiotic susceptibility surveillance to inform antibiotic choice. The antibiotic susceptibility testing was inconsistent as it was not requested at all times by the treating clinician.

Larger, prospective studies are recommended to analyse sepsis biomarkers and determine risk factors for death in paediatric patients with VGS bacteraemia.

### Study limitations and strengths

This study is limited by its retrospective nature and sample size. It was not designed to show causation of disease or powered to analyse risk factors for mortality. In addition, penicillin MIC testing was not performed on all cultures for those infections thought to be clinically significant. Subspecies identification was not carried out for most of the cultures, and this would have been relevant information as some subspecies are more virulent than others. The absence of clinical information for some cases is a further notable factor that limits the interpretation of the data and of sepsis scores. Neonatal sepsis scores were not applied; in retrospect given the proportion of neonates in this cohort, this score may have yielded different results. Despite these limitations, this study has some strengths. Single pure cultures were analysed, and polymicrobial cultures were excluded. This study represents a diverse paediatric patient population with different age groups and includes oncology and non-oncology conditions.

## Conclusion

Viridans Group Streptococci are often viewed as a contaminant when cultured. However, VGS bacteraemia can be associated with serious complications including death. Further prospective studies with larger patient samples are required to be able to determine the significance of VGS in the paediatric setting.
